# Addressing the Evidence Gap in the Economic and Social Benefits of Civil Registration and Vital Statistics Systems: A Systematic Review

**DOI:** 10.3389/phrs.2022.1604560

**Published:** 2022-07-08

**Authors:** Rebeca Revenga Becedas, Carmen Sant Fruchtman, Irina Dincu, Donald De Savigny, Daniel Cobos Muñoz

**Affiliations:** ^1^ Epidemiology and Public Health Department, Swiss Tropical and Public Health Institute (Swiss TPH), Basel, Switzerland; ^2^ University of Basel, Basel, Switzerland; ^3^ Centre of Excellence for CRVS Systems, International Development Research Centre, Ottawa, ON, Canada

**Keywords:** civil registration and vital statistics system, civil registration, vital statistics, birth registration/certification, death registration/certification, marriage registration/certification, divorce registration/certification

## Abstract

**Objectives:** Considering the aspiration embedded in the Sustainable Development Goals to Leave No One Behind by 2030, civil registration and vital statistics systems have an essential role in providing reliable, up-to-date information to monitor the progress. Thus, the aim of this systematic review is to compile empirical evidence on the benefits of a functioning civil registration and vital statistics system.

**Methods:** Selected databases were systematically searched until 2019. Key experts were also contacted for relevant literature. The review process was managed with the software EPPI-Reviewer and followed standard methods for systematic reviews.

**Results:** A total of 18 studies were included. The findings revealed that having birth, death, and/or marriage registration, and vital statistics were associated with access to rights and protection, positive impact on economic and health outcomes, and increased access to education.

**Conclusion:** The present review supports the idea that systemic approaches strengthen civil registration and vital statistics systems due to the cumulative effects of vital events’ registration. Ensuring appropriate systems for civil registration will have an impact not only on the individuals but also on the generations to come.

## Introduction

The 2030 Agenda for Sustainable Development represents a global effort to create a more equitable and sustainable world and it is comprised of seventeen goals referred to as the Sustainable Development Goals (SDGs) [1]. They set out the vital importance of providing legal identity for all, including birth registration by 2030 [2]. Aside from the fact that civil registration and vital statistics (CRVS) systems are a target in themselves, they play a key role in supporting and monitoring the progress towards many of the other SDGs targets [3]. Considering the commitment to meet the global aspiration to leave no one behind (LNOB) [4], a strong CRVS system is the most reliable source of up-to-date and continuous information.

Some of the benefits of a robust CRVS system are well documented [4], although knowledge gaps remain in this area. Unlike other data sources, a functioning CRVS system generates precise and up-to-date demographic and health indicators, providing a continuous stream of vital statistics for local administrative areas. CRVS data can be used by decisions-makers in any sector to detect areas of population change and reallocate resources as needed [5]. Within the context of COVID-19 pandemic, the importance of having a system which can reliably and continuously track fertility rates, mortality rates, and cause-of-death distribution to support public health decisions has been highlighted [6, 7]. Countries with well-functioning CRVS systems are able to produce timely and locally relevant mortality data that permit decision-makers to track mortality, for example due to HIV/AIDS or SARS-CoV-2 [8]. CRVS systems are being positioned as one of the essential services in the response to this health challenge, not only because they can provide information on the number and cause-of-death by age, sex and location, but because they can be also used to provide legal identification when needed to access health services [9].

In addition, CRVS systems also collect information on marriages, divorces, and adoptions [5]. Data on those vital events completes the demographic profile of countries and form the statistical foundation to understand citizens’ needs and design effective targeted policies [2, 10].

### Research Gap

Despite the increased interest in strengthening CRVS systems, progress has been slow in most regions, and especially so in low- and middle-income countries (LMICs) [11]. Although the body of evidence around CRVS systems is considerable and continues to grow, it focuses mostly on how to strengthen the CRVS systems. Most research efforts have primarily looked at intermediate outcomes such as increases in birth registration, barriers to registration, and others.

However, to our knowledge, the effects of having a functioning system on individuals, in particular women, girls, and communities, as well as the mechanisms that underpin their impact, have not been identified and assessed in most settings.

To compile empirical evidence on the benefits of a functioning CRVS system, we have searched and reviewed all peer-reviewed and grey literature assessing the effects of the products of a functioning CRVS system (e.g., birth, death, marriage certificate or vital statistics) on individuals, communities or societies.

## Methods

This systematic review was conducted following standard methodology described elsewhere [12], using a predefined protocol (International Prospective Register of Systematic Reviews identification number CRD42021226169), and following the PRISMA criteria for reporting of systematic reviews [13] (Supplementary File S2).

We reviewed peer-reviewed literature and grey literature as far as they met the inclusion criteria.

### Search Strategy

General and grey literature databases were systematically searched for articles which examine the benefits from CRVS systems. No geographical location or date limitation was applied. CENTRAL, MEDLINE, EMBASE, PubMed, Econlit, Scopus, ELDIS, WHO, WHO IMSEAR and IndMED literature databases were systematically searched until June 2019 (Supplementary File S3). A manual search was performed within web-based databases and repositories of relevant institutions. Also, we approached key experts in the field and did a snowball search on the references of key publications was performed.

### Eligibility Criteria

Eligibility criteria were based on key study features: population, intervention, comparator, outcome, and design, as recommended by the PRISMA group [14]. Studies were included if:

(1) Population: Included any type of population;
(2) Intervention: Studies analysing the effect of the output of CRVS systems (e.g., benefits of registering a death). We excluded studies assessing the effect of interventions aimed at increasing the performance of CRVS systems (e.g., effect of interventions increasing registration completeness);
(3) Comparator: Studies with any comparator which could include the lack of new systems;
(4) Outcomes: Studies reporting quantitative and qualitative outcomes representing the benefit of CRVS system individuals (e.g. improved health outcomes); or societies including governments (e.g., improved governance, reduced fraud);
(5) Design: All types of study design in where methodology was described were included; and
(6) Written in English, Spanish, Portuguese or French.

The integration of quantitative and qualitative evidence is achieved at the level of the design. We applied a data-based convergent synthesis design, addressing one review question [15, 16]. The search strategy was designed by an independent information specialist, in consultations with the review team.

### Selection of Studies

All types of study design examining the benefits from CRVS systems were included. The articles were identified based on the search strategy. The review process was managed with the online software EPPI-Reviewer engine^®^, utilising machine learning to support the screening process [17]. Duplicate references were removed. Two reviewers screened the selected records according to the inclusion and exclusion criteria following the Cochrane Handbook [12]. Any disagreement was resolved by consensus. Articles that met all inclusion criteria were included for quality assessment, using Mixed Methods Appraisal Tool (MMAT) [18], and data extraction. Methodological quality was not used to exclude studies. The included studies were scanned to identify additional literature.

### Data Extraction and Synthesis

Two reviewers completed the data extraction using a standardized template, including general information on the studies, setting, design, and quantitative and qualitative results. Data were categorised according to the type of effect described. The categorization of the effects was based on the themes emerging from the data extracted from the studies.

Quotes referring to benefits of CRVS systems were extracted from qualitative studies. Data extracted from quantitative studies included frequency and effect measures of CRVS systems’ products on specific outcomes, if available.

A gender and equity lens was applied when extracting the data. We assessed whether social characteristics, such as gender/sex, age, ethnicity, among others, were incorporated in the studies to understand how they played a role in contributing to inequities in the access to the benefits of a CRVS system.

Furthermore, we applied a narrative approach to synthesize the findings of the qualitative and quantitative evidence [15]. First, a preliminary synthesis of results was conducted, in form of a thematic analysis. During the process, the studies which focussed on the same outcomes were clustered. Relationships between the results on the different studies were analysed and the final cluster results were refined.

## Results

**FIGURE 1:**
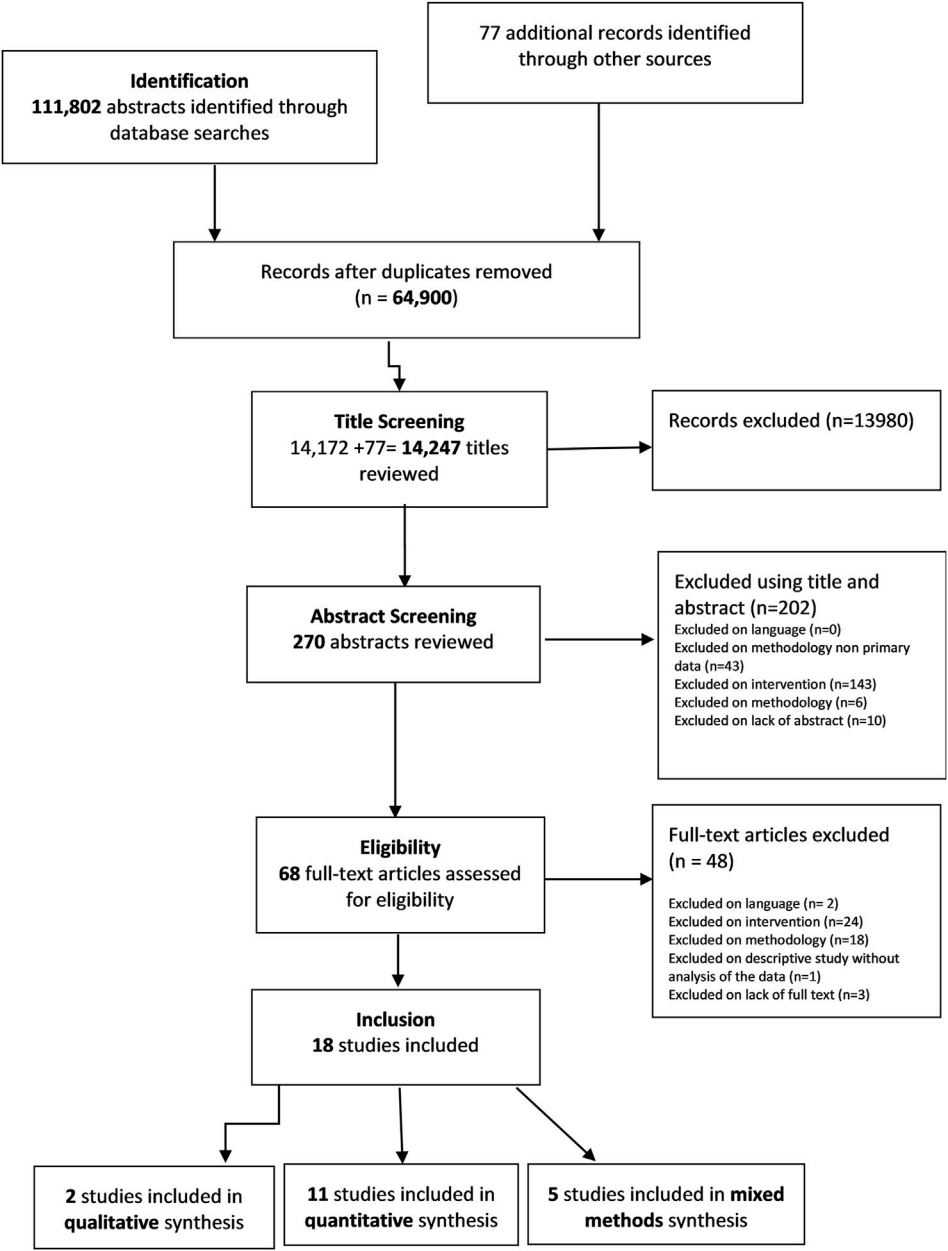
Flow diagram of article selection. Addressing the evidence gap in the economic and social benefits of Civil Registration and Vital Statistics Systems: A Systematic Review, 2021. Adapted from PRISMA (Systematic review, Asia, America, Africa and Europe, 1910–2019).

The screening and selection process is shown in Figure 1. We identified 111,803 records to review. Additional 77 additional studies were retrieved as a result of the inputs from key experts. A total of 18 studies met the eligibility criteria and were included for quality appraisal and data extraction. These included: two qualitative studies, 11 quantitative studies and five mixed methods studies conducted across four continents (Asia, America, Africa and Europe). The main characteristics of the studies can be seen in Table 1. Quantitative results are shown in Table 2.

**TABLE 1:** Characteristics of the 18 studies included in the systematic review. Addressing the evidence gap in the economic and social benefits of Civil Registration and Vital Statistics Systems: A Systematic Review, 2021 (Systematic review, Asia, America, Africa and Europe, 1910–2019).

	References	Location	Year of data collection	Study type	Study population	CRVS output
Birth Registration and Children’s Rights: A Complex Story	[19]	India, Kenya, Sierra Leone, Vietnam	2005–2012	Mixed methods	Children in rural and urban areas	Birth certificate and/or registration
Birth Registration and Protection for Children of Transnational Labor Migrants in Indonesia	[22]	Indonesia	2014	Qualitative	22 families, and 54 adults, children aged 9–14 years in rural areas	Birth certificate and/or registration
Identifying the Rich: Civil Registration and State- Building in Tanzania	[25]	Tanzania	2008–2015	Quantitative	4,000 households	Birth certificate and/or registration
Does birth under-registration reduce childhood immunization? Evidence from the Dominican Republic	[31]	Dominican Republic	2007	Quantitative	Children under 59 months of age	Birth certificate and/or registration
Underlying dynamics of child birth registration in Zimbabwe	[24]	Zimbabwe	2014	Mixed methods	Children—parents/guardian in 105 households in Bindura district	Birth certificate and/or registration
Birth registration and child undernutrition in sub-Saharan Africa	[30]	Thirty-seven sub-Saharan African countries	2014	Quantitative	Children under 5 years of age	Birth certificate and/or registration
Birth Registration and the Impact on Educational Attainment	[28]	Dominican Republic	2007	Quantitative	Children under 5 years of age	Birth certificate and/or registration
Papers, please! The effect of birth registration on child labor and education in early 20th century United States	[21]	United States	1910–1930	Quantitative	Children from 12 to 15 years old	Birth certificate and/or registration
Protection through Proof of Age. Birth Registration and Child Labor in Early 20th Century United States	[20]	United States	1910–1930	Quantitative	Children from 12 to 15 years old	Birth certificate and/or registration
Who Says I Do: The Changing Context of Marriage and Health and Quality of Life for LGBT Older Adults	[32]	United States	2014	Quantitative	LGBT Older Adults	Marriage certificate and/or registration
Associations between birth registration and early child growth and development: evidence from 31 low- and middle-income countries	[29]	31 LMICs	2010–2014	Quantitative	Children aged 36–59 months	Birth certificate and/or registration
Back to what counts: Birth and death in Indonesia. Jakarta, Indonesia	[27]	Indonesia	2015–2016	Mixed methods	5,552 individuals	Birth certificate and/or registration
					1,222 adults (95.7% female and 4.3% male) and 2,361 children (50.4% female and 49.6% male)	Death certificate and/or registration
						Marriage certificate and/or registration
Data for the Sustainable Development Goals: Metrics for Evaluating Civil Registration and Vital Statistics Systems Data Relevance and Production Capacity, Illustrations with Nigeria	[35]	Nigeria	2003, 2008, 2013	Qualitative	CRVS data	Vital statistics
Integrated human rights and poverty eradication strategy: the case of civil registration rights in Zimbabwe	[26]	Zimbabwe	2005–2006	Mixed methods	Individuals who were 13 years and older without any other restriction in their socio-demographic factors	Birth certificate and/or registration
Are well functioning civil registration and vital statistics systems associated with better health outcomes?	[10]	144 countries	2010	Quantitative	CRVS performance data from 144 countries	Vital statistics
Difference-in-Differences Analysis of the Association Between State Same-Sex Marriage Policies and Adolescent Suicide Attempts	[33]	United States	1999–2015	Quantitative	Adolescents who participated in the Youth Risk Behavior Surveillance System in 47 states	Marriage certificate and/or registration
Impact of Civil Marriage Recognition for Long-Term Same-Sex Couples	[34]	United States	2013	Quantitative	Adults who identify as members of female, male same-sex couples	Marriage certificate and/or registration
The work of inscription: antenatal care, birth documents, and Shan migrant women in Chiang Mai	[23]	Thailand	2010–2012	Mixed methods	Shan migrant women from Myanmar in Chiang Mai	Birth certificate and/or registration

**TABLE 2:** Quantitative results extracted from the 18 studies included in the systematic review. Addressing the evidence gap in the economic and social benefits of Civil Registration and Vital Statistics Systems: A Systematic Review, 2021 (Systematic review, Asia, America, Africa and Europe, 1910–2019).

Effect	Indicator (references)	Exposure	Outcome	Result control group	Measure of effect	Results as reported by authors
Access to civil and social rights and services						
Child Labour Elimination	Likelihood that child reports an occupation for individuals aged 12–15 [21]	Being born in a state with a child labour law but without a birth registration law	Child report an occupation		*β* = -0.034	When born before registration law, children below the minimum age were 3.4% points less likely to work than work eligible children
					SE (0.008)	
					*p* < 0.01	
	Likelihood that child reports an occupation for male individuals aged 12–15 [21]	Being born in a state with a child labour law and a birth registration law	Child report an occupation		*β* = 0.09	Male individuals below the minimum aged, were 9% points less likely to work than the work-eligible than the work-eligible, when born with a birth registration law in place
					SE (0.003)	
					*p* < 0.01	
	Likelihood that child reports an occupation for black individuals aged 12–15 [21]	Being born in a state with a child labour law and a birth registration law	Child report an occupation		*β* = 0.078	Black individuals below the minimum age were 7.8% points less likely to work than the work-eligible than the work-eligible, when born with a birth registration law in place
					SE (0.003)	
					*p* < 0.01	
	Likelihood that child reports an occupation in an urban area for individuals aged 12–15 [20]	Being born in a state without a birth registration law in an urban area	Child report an occupation		*β* = -0.028	In urban areas, under-aged children born before the registration laws were 2.8% points less likely to work
					SE (0.008)	
					*p* < 0.01	
	Likelihood that child reports an occupation in a rural area for individuals aged 12–15 [20]	Being born in a state without a birth registration law in a rural area	Child report an occupation		*β* = −0.016	In rural areas, under-aged children born before the registration laws were 1.6% points less likely to work
					SE (0.007)	
					*p* < 0.05	
	Likelihood that child reports an occupation in an agricultural county for individuals aged 12–15 [20]	Being born in an agricultural county with a birth registration law	Child report an occupation		*β* = −0.018	In agricultural counties, children below the minimum age limit were 3.6 percentage points less likely to work if they were born with birth registration laws
					SE (0.010)	
	Likelihood that child reports an occupation in a non-agricultural county for individuals aged 12–15 [20]	Being born in an agricultural county with a birth registration law	Child report an occupation		*β* = −0.028	In non-agricultural counties, children below the minimum age limit were 5.9 percentage points less likely to work if they were born with birth registration laws
					SE (0.007)	
					*p* < 0.01	
Access to protection services	Relationship correlating the access to Basic Education Assistance Module (BEAM) to birth certificate in Zimbabwe [24]	Having a birth certificate	Access to BEAM		*p* = 0.03	The expansion of BEAM and other conditional cash transfers will likely reinforce parents’ and guardians’ perception of direct material benefits of child birth registration. In fact, the study found a significant relationship (*p* = 0.03) between access to beam and possession of birth certificates
Positive impact on economic outcomes for individuals and governments						
Increased the amount of tax payers	Likelihood that registered citizens paid council tax in Tanzania [25]	Having a birth registration	Tax outcomes		*β* = 0.229	In one of the reform districts, registered citizens are 22.9 percentage points more likely to pay council taxes
					SE (0.099)	
					*p* < 0.05	
Increased the access to formal economic sector	Probability of having a bank account in Tanzania [25]	Having a birth registration	Finance outcomes		*β* = 0.477	Birth registration is associated with a 47.7% point increase in the probability of someone in the respondent’s household possessing a bank account
					SE (0.244)	
					*p* < 0.05	
Human capital for economic development: Increased access to education and educational attainment						
Increased access to education	Probability of being enrolled in formal education in India [19]	Having a birth certificate	Attending formal education		*β* = 0.6368	A sponsored child with birth registration is 37% more likely to be attending formal education in India
					SE (0.156)	
					OR = 1.890	
					*p* < 0.05	
	Probability of being enrolled in formal education in Kenya [19]	Having a birth certificate	Attending formal education		Data not included	A sponsored child with birth registration is 50% more likely to be attending formal education in Kenya
	Probability of being enrolled in formal education in Sierra Leone [19]	Having a birth certificate	Attending formal education		Data not included	A sponsored child with birth registration is 60% more likely to be attending formal education in Sierra Leone
	Likelihood that child attends school for individuals aged 12–15 [21]	Being born in a state with a child labour law but without a birth registration law	Child school attendance		*β* = 0.036	Under-aged children born before the registration law were 3.6 percentage points more likely to attend school than the work-eligible
					SE (0.008)	
					*p* < 0.01	
	Likelihood that child attends school for individuals aged 12–15 [21]	Being born in a state with a child labour law but with a birth registration law	Child school attendance		Data not included	Those born in a state with a birth registration law were 6.5 percentage points more likely to attend school than the work-eligible
	Likelihood that school-age children were enrolled in Indonesia [27]	Having a birth certificate	Enrolled in school		AOR = 2.0, 95% CI = 1.6–2.5	School-age children that were enrolled at the time of the study were twice as likely to have a birth certificate as those who were not enrolled in school
Increased educational attainment	Likelihood that children aged 11 to 18 pass the first cycle primary school in Dominic Republic [28]	Lack of birth certificate	Passing the first cycle primary school		OLS	An unregistered child would have between 20 and 40-% points lower probability of passing the first cycle of primary school
					*β* = −0.227	
					SE = 0.084	
					*p* < 0.01	
					PROBIT	
					*β* = −0.393	
					SE (0.217)	
					*p* < 0.1	
	Years of education in 1960 (birth cohorts: 1896–1925), OLS [21]	Being born in a state with birth registration laws	Education		*β* = 0.083	For individuals born in the United States between 1896 and 1925, the average educational attainment increased from 8.7 to 11 years
					SE (0.21)	
					*p* < 0.01	
					*β* = 0.103	The coverage of the registration law increased from 25 to 100% for the same cohorts. Thus, a 0.09 years increase in attainment due to the birth registration laws would account for 3% of the total increase
					SE (0.31)	
					*p* < 0.01	
	Probability of English literacy as an education outcome in Tanzania [25]	Having a birth certificate	Education		*β* = 0.411	This effect on the population of compliers, birth registration is associated with a 41-percentage point increase in the probability of English literacy
					SE = 0.185	
					*p* < 0.05	
	Likelihood that adults attended elementary or middle school in Indonesia [27]	Having a birth certificate	Attended elementary or middle school in Indonesia		AOR = 2.1, 95% CI = 1.1–3.8	Adults that had attended elementary or middle school were twice as likely to have a birth certificate as those who had not attended school
	Likelihood that adults attended high school or higher in Indonesia [27]	Having a birth certificate	Attended high school or higher		AOR = 3.7, 95% CI = 1.9–7.2	Adults that had attended high school or higher were almost four times as like to have a birth certificate than those who had not attended school
General	Likelihood that Healthy Life Expectancy (HALE), in 144 countries [10]	Associated to vital statistics performance index (VSPI)	HALE		*β* = 1.044	HALE was estimated to increase by 0.044% with each unit increase in VSPI on a 100-point scale
					95% CI [1.020–1.068]	The regression indicates that if worldwide CRVS performance was high (0.9) rather than at its worldwide average based on the 144 countries or territories with available data (0.591), average HALE would increase by nearly 1 year (63.1 years vs. 62.3 years)
					*p* < 0.0005	
	Likelihood that Maternal Mortality Ratio (MMR) in 144 countries [10]	Associated to vital statistics performance index (VSPI)	MMR		*β* = 0.721	Countries with high VSPI values have low MMR.
					95% CI	
					0.508–1.023	
					*p* = 0.067	
	Likelihood that child mortality risk (5q0) values, in 144 countries [10]	Associated to vital statistics performance index (VSPI)	5q0		*β* = 0.418 0.367–0.470	Countries with high VSPI values have low 5q0
					*p* = 0.002	
Improved nutrition	Values of children’s height-for-age Z-score (HAZ) in 40 cases out of 140 comparisons [30]	Registered vs. not registered children	HAZ		*p* < 0·1	Effects of selection bias due to birth registration on undernutrition prevalence
			28.6%			Registered children generally presented a better nutritional status than unregistered ones, with significantly higher HAZ mean values in 40 cases out of 140 comparisons: 28.6%
	Values of children’s Weight for age Z-score (WAZ) in 51 cases of 140 comparisons [30]	Registered vs. not registered children	WAZ		*p* < 0·1	Effects of selection bias due to birth registration on undernutrition prevalence
			36.4%			Registered children generally presented a better nutritional status than unregistered ones, with significantly higher WAZ mean values in 51 cases out of 140 comparisons: 36.4%
	Values of children’s Weight-for-height Z-score (WHZ) <–2) in 38 cases of 140 comparisons [30]	Registered vs. not registered children	WHZ		*p* < 0·1	Effects of selection bias due to birth registration on undernutrition prevalence
			27.1%			Registered children generally presented a better nutritional status than unregistered ones, with significantly higher WHZ mean values in 38 cases out of 140 comparisons: 27.1%
	Likelihood that children are stunting in Uttar Pradesh, India [19]	Having a birth registration	Nutrition outcomes		*β* = -0.073	In Uttar Pradesh a child with birth registration is approximately 0.7 times less likely to be stunted
					SE (0.019)	
					OR [0.670]	
					*p* < 0.001	
	Likelihood that children are under-weight in Uttar Pradesh, India [19]	Having a birth registration	Nutrition outcomes		*β* = -0.0423	In Uttar Pradesh a child with birth registration is approximately 0.8 times less likely to be under-weight
					SE (0.018)	
					OR [0.781]	
					*p* < 0.05	
	Likelihood that children are stunting in Kenya [19]	Having a birth registration	Nutrition outcome		*β* = -0.0548	In Kenya a child with birth registration is approximately 0.8 times less likely to be stunted
					SE (0.023)	
					OR [0.767]	
					*p* < 0.005	
	Likelihood that children are under-weight in Kenya [19]	Having a birth registration	Nutrition outcome		*β* = -0.00712	In Kenya a child with birth registration is approximately 0.7 times less likely to be under-weight
					SE (0.021)	
					OR [0.658]	
					*p* < 0.001	
	Likelihood of children’s height-for-age z-scores (HAZ) aged 36–59 months in 31 LMICs [29]	Lack of birth certificate	Nutrition outcome		*β* = −0.18	Not having a birth certificate was negatively associated with children’s HAZ
					95% CI: −0.23, −0.14	
					*p* < 0.001	
	Likelihood of children’s weight-for-age z-scores (WAZ) aged 36–59 months in 31 LMICs [29]	Lack of birth certificate	Nutrition outcome		*β* = − 0.10	Not having a birth certificate was negatively associated with children’s WAZ
					95% CI: −0.13, −0.07	
					*p* < 0.001	
	Likelihood of children’s ECDI z-score aged 36–59 months in 31 LMICs [29]	Lack of birth certificate	Nutrition outcome		*β* = −0.10	Not having a birth certificate was negatively associated with children’s ECDI z-scores
					95% CI: −0.13, −0.07	
					*p* < 0.001	
Better vaccination outcomes	Effect on number of vaccines for children aged 0–59 months in Dominic Republic [31]	Lack of birth certificate	Immunisation		*β* = -0.755	This estimate suggests that lacking a birth certificate is associated with a reduction of 0.7 vaccines
					SE (0.156)	
					*p* < 0.01	
	Effect on vaccination outcomes in Uttar Pradesh, India [19]	Having a birth registration	Immunisation		BCG	Children with birth registration in Maharashtra, India are between 1.2 and 3.8 times more likely to have been vaccinated than children without birth registration, depending on the type of vaccine (e.g., BCG, POL0, DPT1, DPT2, DPT3, Measles, POL1)
					SE (0.031)	
					OR [2.127]	
					POL 0	
					SE (0.032)	
					OR [1.898]	
					DPT1	
					SE (0.031)	
					OR [2.092]	
					DPT2	
					SE (0.033)	
					OR [2.012]	
					DPT3	
					SE (0.033)	
					OR [1.815]	
					Measles	
					SE (0.033)	
					OR [2.006]	
					*p* < 0.01	
					—	
					POL 1	
					SE (0.016) [1.805]	
					*p* < 0.1	
	Effect on vaccination outcomes in Kenya [19]	Having a birth registration	Immunisation		BCG	Children with birth registration in Kenya are between 1.3 and 2.2 times more likely to have been vaccinated than children without birth registration, depending on the type of vaccine (e.g. BCG, POL0, DPT1, DPT2, DPT3, Measles, POL1)
					SE (0.014)	
					OR [2.235]	
					POL 0 (0.019) [1.807]	
					DPT1 (0.015) [1.887]	
					POL 1 (0.015) [1.901]	
					DPT2 (0.017) [1.866]	
					POL 2 (0.017) [1.700]	
					DPT3 (0.019) [1.469]	
					POL 3 (0.020) [1.364]	
					Measles (0.020) [1.484]	
					*p* < 0.01	
	Effect on vaccination outcomes in Sierra Leone [19]	Having a birth registration	Immunisation		BCG	Children with birth registration in Sierra Leone are between 1.6 and 2.1 times more likely to have been vaccinated than children without birth registration, depending on the type of vaccine (e.g. BCG, POL0, DPT1, DPT2, DPT3, Measles, POL1)
					SE (0.016)	
					OR [2.126]	
					POL0 (0.020) [1.658]	
					*p* < 0.05	
					—	
					DPT1 (0.018) [1.845]	
					POL 1 (0.018) [1.948]	
					DPT2 (0.019) [1.767]	
					POL 2 (0.019) [1.797]	
					DPT3 (0.019) [1.711]	
					Measles (0.019) [1.589]	
					*p* < 0.01	
Improved mental health and quality of life	Changes in high school student (ages 15–19) suicide attempts in US. Net Change in Suicide Attempts, Percentage Points [33]	After implementation of Same-Sex Marriage Policies	Suicide attempts- (Students identifying as sexual minorities) –4.0	(All students) −0.6	95% CI	The reduction in suicide attempts, represents a 7% relative reduction in the proportion of high school students attempting suicide owing to same-sex marriage implementation
					–6.9 to –1.2	
					*p* < 0.01	
					—	
					95% CI	These results are equivalent to a 14% relative decline in the proportion of adolescents who were sexual minorities reporting suicide attempts in the past year
					–1.2 to –0.01	
					*p* < 0.05	
	Positive LGBIS identity and social support outcomes by marital status and state recognition [34]	Having a marriage registration	LGB Identity centrality		95%	The positive coefficients for marital status indicate that individuals who are in a civil marriage report significantly higher levels of LGB identity centrality
			γ020 = 0.20		CI = [0.04, 0.36]	
					*p* < 0.05	
	Positive LGBIS identity and social support outcomes by marital status and state recognition [34]	Having a marriage registration	Social support from partner γ020 = 0.17		95%	The positive coefficients for marital status indicate that individuals who are in a civil marriage perceive their partner as more supportive
					CI = [0.04, 0.30]	
					*p* < 0.01	
	Mean/(SE) or %—general health and quality of life (QOL) characteristics by relationship status and gender, between married and unmarried partnered men in 2017, United States [32]	Having a marriage registration	General Health married men	unmarried partnered men	*p* < 0.05	Married men showed advantages over unmarried partnered men in general health -QOL
			3.62/(0.10)	3.29/(0.11)		
	Mean/(SE) or %—physical health and quality of life (QOL) characteristics by relationship status and gender, between married and unmarried partnered men in 2017, United States [32]	Having a marriage registration	Physical Health married men	unmarried partnered men	*p* < 0.05	Married men showed advantages over unmarried partnered men in physical health -QOL
			77.05/(1.39)	69.58/(2.45)		
	Mean/(SE) or %—environmental health and quality of life (QOL) characteristics by relationship status and gender between married and unmarried partnered men in 2017, United States [32]	Having a marriage registration	Environmental Health married men	Unmarried partnered men	*p* < 0.05	Married men showed advantages over unmarried partnered men in environmental health -QOL
			82.51/(1.39)	75.72/(1.45)		
	Mean/(SE) or %—general health characteristics by relationship status and gender—quality of life (QOL) between married and single women in 2017, United States [32]	Having a marriage registration	General Health married women	Single women	*p* < 0.05	Married women showed advantages over single women in general health -QOL
			3.64/(0.10)	2.88/(010)		
	Mean/(SE) or %—psychological health -quality of life (QOL) characteristics by relationship status and gender between married and single women in 2017, United States [32]	Having a marriage registration	Psychological	Single women	*p* < 0.05	Among women, those who were legally married had better general health, lower rates of disability, and better QOL across all domains compared with those who were single
			Health married women	62.22 (2.05)		
			71.64 (1.42)			
	Mean/(SE) or %—social health and quality of life (QOL) characteristics by relationship status and gender between married and unmarried women in 2017, United States [32]	Having a marriage registration	Social	Unmarried women	*p* < 0.05	Married women only had greater social QOL than those unmarried partnered
			Health married women	63.39 (2.49)		
			72.85 (1.87)			

**FIGURE 2:**
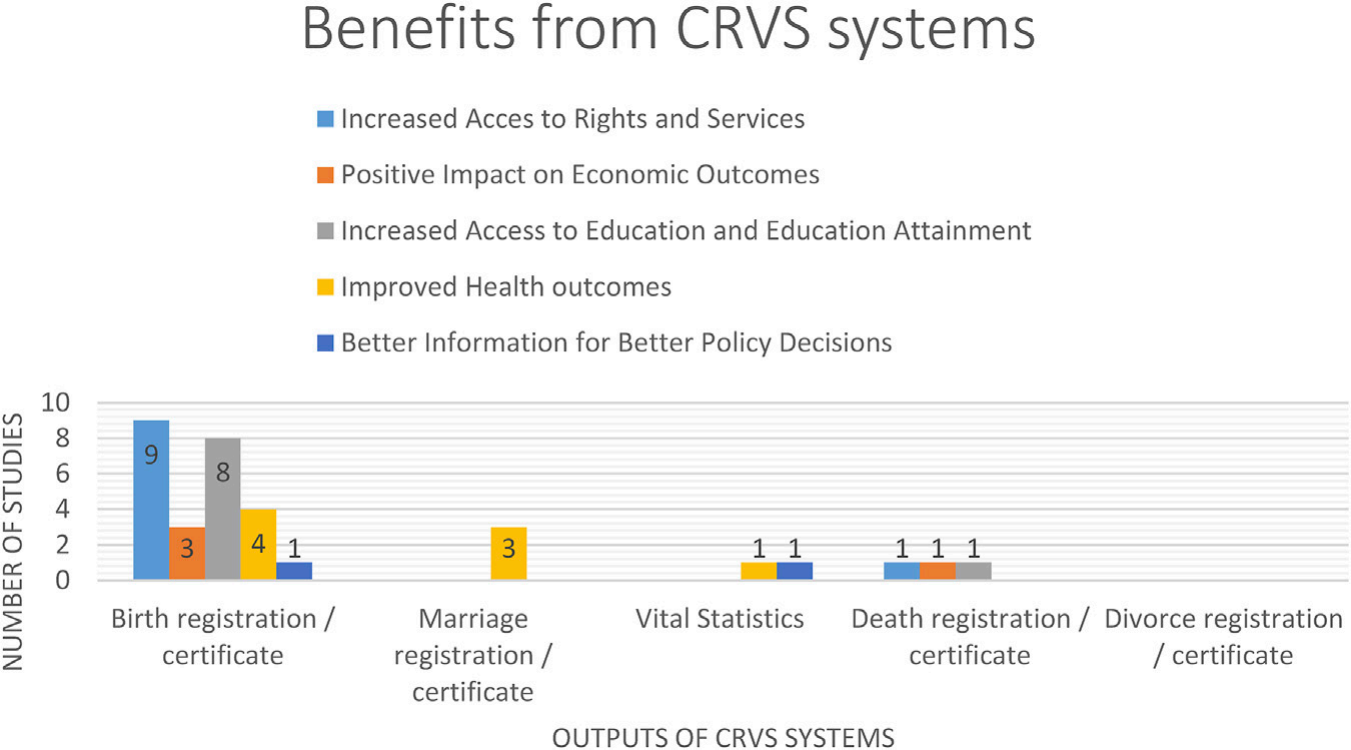
Table Chart of the benefits of CRVS systems per vital event. Addressing the evidence gap in the economic and social benefits of Civil Registration and Vital Statistics Systems: A Systematic Review, 2021 (Systematic review, Asia, America, Africa and Europe, 1910–2019).

We report the results of the studies in six sections: 1) access to civil and social rights and services, 2) positive impact on economic outcomes for individuals and governments, 3) human capital for economic development: increased access to education and education attainment, 4) improved health outcomes, 5) better information for better policy decisions. The benefits from each vital event are shown in Figure 2.

The two qualitative and 11 quantitative studies achieved a moderate methodological quality, while the mixed-methods studies were found to be of lower quality due to the quantitative components. The poorest scoring items on the MMAT were the questions related to the sampling strategy and the risk of nonresponse bias. All the studies had a clear study question and presented the results with clarity (Supplementary File S1).

### Access to Civil and Social Rights and Services

The links of vital registration to other human and civil rights have been extensively investigated in the included studies. Ten out of eighteen studies focused on the effect of birth registration on child protection and access to services, specifically the prevention of child labour and access to legal identity documents.

Having proof of age is not only an essential element for governments to run protection programs but also to prevent child labour and child marriage through the enforcement of laws [19–21]. It has been also seen as a way to defend them in situations where their rights have been violated.

“If you don’t have a birth certificate, and you are abused, no one will know if you are a child or not, so they can’t do anything.” (a Plan program staff in urban Sierra Leone) [19].

A study suggested that child labour laws and schooling laws implemented between 1910 and 1930 in the United States reduced under-aged employment and raised the school attendance of school-aged children [21]. The effect was also seen across ethnicities [21], and geographical location [20]. Results were consistent with those reported in India, Kenya and Sierra Leone [19].

Another group of studies looked at the impact of vital registration on migrant populations. In Indonesia [22], Sierra Leone [19], and Thailand [23], authors concluded that birth registration is a pathway to access the education and obtain legal identity documents. As a consequence, it increases the likelihood for those individuals to access better economic opportunities. In Thailand, it was reported that having a birth certificate gives rights and citizenship to migrant children from Myanmar [23].

“When he reaches 15 years, he can apply for a Thai identification card, because we already have both his birth registration and have prepared all the documents required of him (…).” (Shan woman in her mid-30s) [23].

In Zimbabwe, the function of a birth certificate was underlined as documentary proof of family and kinship by each respondent in the study.

“(…) a vehicle for establishing blood ties to a father, family and kinship group whilst validating one’s nationality and citizenship.” [24].

Access to social protection programs, such as conditional cash transfer programs, food subsidies or education support is not possible without a birth certificate in many countries. In Zimbabwe, participants in a study reported that the birth certificate as a way to identify children and allow them to access cash transfers and school feeding schemes [24]. In Vietnam, being registered to one specific household was essential to have any social protection from the state, which could have a lifetime effect [19].

“(…). Birth certificates are very useful in so many cases in Vietnam: even when people become 80 years old they will need a birth certificate to get a pension” (parents in a FGD, Vietnam) [19].

In Tanzania, a study showed the positive effect of birth registration on gaining access to social security systems, formal employment, property rights and better house quality [25].

### Positive Impact on Economic Outcomes for Individuals and Governments

Results of three studies highlighted the association between birth registration to increased access to formal labour employment, banking and probable improvement in the individual’s socioeconomic status. On the government side, having a functioning CRVS system was associated with increased tax revenue, improved administrative and governance processes and targeting social and economic subsidies and incentives.

In Tanzania, researchers concluded that citizens who were registered were 22.9% points more likely to pay council taxes. A positive relationship was shown between having a birth certificate and working in the formal private sector as a paid employee. The study also showed a 47.7% point increase in the probability of having a bank account [25]. The authors stressed the risks of using the argument of the increase in tax revenue as this could lead to target only richer segments of the population in CRVS strengthening efforts which would perpetuate structural inequities in the population [25].

In a multi-country study, it was claimed that having a birth certificate not only improved access to the formal sector but also to jobs that are better paid and stable within the Government or large companies [19].

“(…). For the Government jobs they want them. They want to know your age so that they can know when you are going to retire.” (family’s interview, in India) [19].

“If you are working for the Government, or if you have one of those office jobs.” (mother, in Sierra Leone) [19].

In Zimbabwe, authors explored the impact of having civil registration documents, particularly birth registration, on the poverty eradication strategy. It was found that citizens had to make eight to ten attempts to collect the documents and that most of the respondents were affected by the lack of parent’s certificates. Nevertheless, 81.4% of citizens stated that they would still go through the process again due to the attached importance [26].

The benefit of having a death certificate was explored in a study in Indonesia. Respondents highlighted the importance of this certificate in order to claim the spouse’s pension (10.6%), or grief compensation allowance or inheritances (9.6%), and closing out the bank accounts or health insurances (28.8%). The lack of knowledge of the benefits of having a death certificate (43.8%) was one of the major barriers appointed by the respondents [27].

### Human Capital for Economic Development: Increased Access to Education and Educational Attainment

Nine studies suggested a positive relationship between the possession of a birth certificate and the ability of the child to access education [19, 21, 24, 25, 27]. Children with a birth certificate in India, Kenya and Sierra Leone, were 37%, 50% and 67% more likely to attend school respectively [19]. It was also reported that the lack of a birth certificate did not affect everyone equally. Not only girls reported more gender discrimination experiences, but also, those with privilege could influence the system to obtain a certificate and eliminate barriers.

“…birth certificates are a basic requirement. But if you have the money and influence you can solve anything. Money is given and the rules are easily broken.” (young person, India) [19].

Researchers further supported the idea that birth registration laws were associated with a higher likelihood of children attending school in the United States (6.5% points higher in children born after the registration law) [20, 21]. In Tanzania, having a birth certificate was associated with being more literate, starting school younger and completing more education years (41% points increase in the probability of English literacy) [25].

Further studies also investigated the association between having a birth certificate and the educational attainment of children. In the Dominican Republic, children without a birth certificate had a lower probability of passing the first schooling cycle [28]. In the United States the implementation of the birth registration law increased the average of educational attainment from 8.7 to 11 years and the coverage of the registration law from 25 to 100% for the same cohort [21] and in Zimbabwe respondents believed that the lack of birth registration had negatively affected their education [26].

The interdependency among vital events’ registration was captured in different population groups in Indonesia. Among migrant populations, the lack of a marriage certificate was considered a barrier in registering the birth of a newborn, which has negative consequences during child’s lifetime [22]. Those challenges are reinforced by the existing religious and cultural norms which prevent the most vulnerable groups, such as single mothers, unmarried couples of obtaining legitimate birth registration, those having a parent with a disability, or women working overseas [22].

“A government midwife in one of the villages (…) was ‘not brave enough’ to accept unmarried couples or single mothers as clients due to stigma at the village level against children born out of wedlock. Women who become pregnant out of wedlock in Indonesia or while working overseas cannot get official statements of birth, impeding their access to a legitimate birth certificate for their newborn.” [22].

In Indonesia, adults who attended high school and children in school were almost four-times and twice as likely, respectively, to attain a birth certificate. So, access to education was one of the main reasons families persuaded the certificates for their children [27].

“If they don’t have a [birth certificate], they won’t be able to enrol in school. That’s the point when the communities started to get birth certificates.” (official, Indonesia) [27].

In Zimbabwe and Indonesia, the lack of parental documents affects children’s birth registration [26, 27]. It was noticed that non-registration influences the decision to continue education due to the barriers caused by not having registration documents. It seems that there is a clear connection between the lack of birth registration and early marriage or early employment [26]. This interdependency between the vital events’ registrations affected parents who could not register their marriage due to the lack of their own birth certificates.

“(…) if getting married needs a birth certificate, for those who don’t have one [KUA] won’t take their application (…)” (participant FGD, Indonesia) [27].

From an operational perspective, the dependency among vital events registration means that focusing on just one area of a CRVS system will not yield the full potential of these systems to improve the lives and wellbeing of individuals, including the most neglected.

### Improved Health Outcomes

Eight studies showed a positive association between birth and marriage registration and having vital statistics with nutrition, immunisation, better mental health and quality of life, having a direct effect for women, children and Lesbian, Gay, Bisexual, Transgender, Queer, Intersex (LGBTQI) people.

In a modelling exercise, researchers found a positive relationship between the vital statistics performance index and the national estimates of healthy life expectancy, maternal mortality ratio, and child mortality risk (5q0) in 144 countries [10]. Some further studies have identified birth certificates as the gateway document to access health services or national health insurance programmes. In Vietnam, birth certification was compulsory to obtain a health insurance card [19].

Three studies showed the impact of birth registration on child nutrition and development outcomes. Researchers estimated that the lack of a birth certificate was associated with lower height-for-age z-scores (stunting), weight-for-age z-scores (underweight), and early child development index among children aged 36–59 months using data from 31 LMICs [29]. Similar results were reported in an analysis of multiple indicator cluster surveys from 37 Sub-Saharan countries [30]. In this same study, authors found a potential for selection bias in surveys favouring registered children (or children with a valid date of birth) that could lead to an underestimation of the undernutrition prevalence (up to 28% in some cases). Evidence from India and Kenya supports these findings where children with a birth certificate were less likely to be stunted and underweight [19].

Studies conducted in several countries consistently reported a positive correlation between being registered at birth and vaccination outcomes, in which respondents considered of great importance to know the precise age to provide age-appropriate care [19, 31]. In the Dominican Republic, authors reported that children between 0 and 59 months that did not have birth certificates were behind by nearly one vaccine (out of a total of 9) [31]. In Kenya and India, researchers showed that children had from 1.2 to 3.8 higher chances of being vaccinated, depending on the type of the vaccine, when possessing a birth certificate [19].

A number of studies evaluated the association between civil registration and mental health among LGBTQI people in the United States [32–34]. An analysis of the survey “Aging with Pride” showed that legally married same-sex couples, were advantaged over unmarried partners and singles, having a better general, physical and environmental health [32]. It was showed similar results and confirmed that civil marriage has a positive impact on the wellbeing of individuals in long-term same-sex couples across all the USA states [34]. The association between state same-sex marriage policies and adolescent suicide attempts was investigated in the United States [33]. The application of same-sex marriage policies was associated with a reduction of 0.6% points in suicide attempts among high school students, accounting for a 7% relative reduction in the proportion of high school students who attempted suicide. And the reduction of 4% points in suicide attempts among sexual minorities, equivalent to a 14% relative decline [33].

### Better Information for Better Policy Decisions

Having accurate information about births, deaths and cause-of-death is essential for governments to inform policies. A recent study in Nigeria concluded in their analysis that the national CRVS systems could provide relevant data for up to 25% of the SDGs [35]. However, given the current performance of the system, the CRVS system could not be used to this end. In a multi-country study, Kenya, Sierra Leone, India and Vietnam, government officials stated that accurate data gathered by the civil registration offices is essential at central, district and local levels [19].

“We need to know the region the child comes from. This is important to ensure that resources are allocated correctly. We have some areas that are disadvantaged in the northeast regions. We have a programme that targets these regions—girls are provided for when they go to secondary school” (representative from MoE, Kenya) [19].

The lack of disaggregated data from vital statistics (e.g., sex, age and geographical location) remains critical component in many states. Research has highlighted that disaggregation is a priority for the government to allocate resources appropriately [19].

## Discussion

CRVS systems are complex adaptive systems that perform hundreds of activities daily [36] and impact individuals and societies through multiple channels. Identifying the pathways by which countries can benefit from having a functioning CRVS system is an essential element to advocate for further support to strengthen them. Researchers captured some of these pathways in a model that accounted for some of the complexity in the relationship between CRVS performance and health outcomes. Even though the authors succeeded in generating a robust argument for the effect of CRVS on health, the model was silent about the evidence supporting some of the key assumptions made in the calculations [10]. With this review, we try to fill part of that gap with a systematic analysis of the literature published about the economic and social benefits of CRVS systems.

The evidence included in this review suggests that CRVS systems are enablers for citizens, particularly for women, girls and diverse people (such as people with different ethnicities, gender, sexual orientation, abilities and location), in accessing civil rights [19–24] or social protection programs [19, 24, 25, 27]. A consistent association with improved health [19, 29–34] and education outcomes [19, 21, 24, 25] was also found.

Historical evidence, such as the Shattuck Report on birth and death statistics set the groundwork for the creation of health and social policies in the United States [37]. Further studies demonstrated that vital events registration and certification might result in several society and individual benefits [38, 39]. Also, vital registration creates important data with significant policy utility [39–41]. England and Sweden country cases showed that when a CRVS system is adopted on a systematic basis, certainly led to better health and socioeconomic growth [39].

Further evidence has suggested that birth registration can provide timely information to health decision-makers about the numbers of infants eligible for early childhood vaccinations. Conversely, unregistered children who are brought to vaccination centers can benefit from later or delayed registration. This mutually beneficial relationship is common, especially in settings where vaccination rates and higher than birth registration rates [42–44]. CRVS systems also proved to have a positive impact on economic outcomes for governments since they can enhance the capacity to identify citizens which contribute to increasing tax revenue [45] as well as better administrative and governance procedures [19] and better targeting social and economic subsides and benefits [46].

One of the most striking findings that emerged from this review is that, despite the importance of CRVS systems as a government function [19], there remains a paucity of published evidence on the impact of CRVS systems at any level. We found a significant body of knowledge investigating either interventions to strengthen CRVS systems (e.g., how to increase birth registration) [47], or evaluating their performance [48], but little has been published on the effects of having a birth or death certificate on people’s wellbeing. There is a great potential of such interventions to generate quasi-experimental evidence on the (causal) impact of CRVS systems on the outcomes of interest. When looking at the products form the CRVS system that has been investigated, most of the studies focused on the registration/certification of births. There was limited evidence describing the direct or indirect benefits of having a death registration system in place. Attaining a death certificate was highlighted to be eligible to receive the death benefits, claim insurances or inheritances, as well as cancelling accounts [27]. The need for empirical evidence describing the benefits of death registration data from CRVS systems is essential to enhance the value and increase the government’s incentives to invest in innovation [49].

CRVS systems are especially beneficial for vulnerable groups and neglected communities, including migrant population [19, 23, 24, 27, 50]. The lack of civil registration as a consequence of the interplay between different social factors that excluded at-risk groups from accessing fundamental rights and services, putting them at a higher threat of exclusion [51]. Legal documents produced by CRVS systems such as birth and marriage certificates allow citizens to access other legal documents such as a passport, national ID or voting card [19, 24, 25]. Further literature suggested that women can use a marriage certificate to prove changes in residence or name after marriage when registering to vote [52], as well as governments prevent vote fraud. A birth certificate gives them also a documentary proof of identity and kinship as a way to establish the family ties and track the major events of the life of a child’s life [24, 27]. There is a wealth of documented experiences on the effects of births and marriage registration in preventing child marriage [50, 53, 54], however no empirical research was found. In humanitarian or emergency contexts, children are extremely vulnerable, and the registration of a newborn birth can support family tracing and the entitlement of rights [55].

One interesting finding from this review is that the benefits of the registration/certification of the different vital events are interdependent and have a cumulative impact across the life course of individuals [22, 24, 26]. This argument is consistent with previous evidence, arguing the importance of civil registration for minority groups populations across generations [56–59]. Recent evidence from the Syrian crisis showed how not having verification of marriage may be a bottleneck registering other vital events, which often sustains and amplifies statelessness among children [60, 61]. These findings reinforce the need for systemic approaches to strengthening CRVS systems. Rather than focusing on one vital event, governments need to assure that vital events throughout the entire life course of individuals are registered, certified and included in the vital statistics [62].

Strengthening CRVS systems has become a priority in the global agenda. A large number of countries declared CRVS as essential service worldwide during the COVID-19 pandemic [63–65]. The statistical operations worldwide, including the activities of CRVS systems, has been substantially impacted [63–65]. There is a need to integrate a strategic and systematic approach to support inclusive and sustainable development. However, political will and in-country long term investments are necessary to achieve the current goals. Building links across sectors will increase the stated benefits of CRVS systems, for both the government and its citizens. It will not only avoid fragmentation and increase efficiency, but also facilitate to verify the authentication of people and the occurrence of vital events to claim rights [47, 66]. CRVS systems has the potential to produce timely and relevant statistics [19], and when synchronised with other population data sources, can be mutually-reinforcing. The current crisis underlined the relevance of the digital transformation, while also creating new chances to expand and modernize national statistical agencies [67].

This systematic review is subject to some limitations. First, only studies written four languages were included. We cross-checked previously identified relevant studies to minimise the chances that relevant papers would have been excluded. Second, due to the reduced numbers of studies included, no quantitative sub-analysis was conducted. Selective reporting in both qualitative and quantitative studies could not be ruled out. Third, when matching the inclusion criteria, the methodological quality was moderate to weak, providing vague evidence to show a causal impact. Fourth, this systematic review found evidence of only two of the multiple vital events, such as birth and marriage registration. Including whether the missing interventions had effects on additional outcomes merits further research.

## Conclusion

In conclusion, to our knowledge, this systematic review is the first assessment on the benefits of a functioning CRVS system.

Findings from our systematic review suggested that a strong and accessible CRVS system have a broad range of benefits for individual, communities and governments. Most studies focused on the benefits of registering or certifying vital events such as birth or marriage, as well as vital statistics. The benefits of the registration or certification of death events was appallingly neglected in the literature. This systematic review reinforces the idea of systemic approaches to strengthening CRVS systems due to the cumulative effect found in a number of studies.

There is a need to better document the impact of functioning CRVS systems with robust study designs and that represent different regions and populations. It is also essential to assess the effects of CRVS systems in different population groups using intersectional lens.

Future updates of this review ought to pursue the follow up of the studies stated here, as well as any upcoming investigations.

While this review has not found a large number of studies with a strong scientific methodology demonstrating effects associated with the registration of vital events, the implication for practise and research is that strengthening CRVS systems will increase the benefits for individuals and societies across the life course and across generations, providing governments with information essential for public decision-making.

## Data Availability

The datasets supporting the conclusion of this article are included within the article and its Supplementary Material.

## Supplementary Material

Supplementary File S1 Quality Appraisal. Listing the quality appraisal ratings of each study using Mixed Methods Appraisal Tool.

Supplementary File S2 PRISMA checklist. Addressing the evidence gap in the economic and social benefits of Civil Registration and Vital Statistics Systems: A Systematic Review, 2021. (Systematic review, Asia, America, Africa and Europe, 1910–2019).

Supplementary File S3 Systematic review search strategy. Addressing the evidence gap in the economic and social benefits of Civil Registration and Vital Statistics Systems: A Systematic Review, 2021. (Systematic review, Asia, America, Africa and Europe, 1910–2019).

## Abbreviations

COVID-19, Corona Virus Disease 2019; CRVS, Civil Registration and Vital Statistics; FGD, Focus Group discussions; LGBTQI, Lesbian, Gay, Bisexual, Transgender, Queer, Intersex; LMICs, Low and Middle Income Countries; LNOB, Leave No One Behind; MMAT, Mixed Methods Appraisal Tool; SDGs, Sustainable Development Goals; SwissTPH, Swiss Tropical and Public Health Institute; United States, United States of America; 5q0, Child Mortality Risk.

## References

[1] Resolution GA. Transforming Our World: The 2030 Agenda for Sustainable Development (2015). Geneva: United Nations.

[2] Mills S Abouzahr C Kim J Rassekh BM Sarpong D . Civil Registration and Vital Statistics (CRVS) for Monitoring the Sustainable Development Goals (SDGs). World Bank (2017). Available from: http://documents.worldbank.org/curated/en/979321495190619598/Civil-registration-and-vital-statistics-CRVS-for-monitoring-the-Sustainable-development-goals-SDGS .

[3] Mills S Lee JK Rassekh BM . An Introduction to the Civil Registration and Vital Statistics Systems with Applications in Low-And Middle-Income Countries. J Health Popul Nutr (2019) 38, 23. 10.1186/s41043-019-0177-1 31627735 PMC6800489

[4] Mikkelsen L Phillips DE AbouZahr C Setel PW de Savigny D Lozano R A Global Assessment of Civil Registration and Vital Statistics Systems: Monitoring Data Quality and Progress. Lancet (2015) 386(10001):1395–406. 10.1016/S0140-6736(15)60171-4 25971218

[5] AbouZahr C Mathenge G Brøndsted Sejersen T Macfarlane SB . Civil Registration and Vital Statistics: A Unique Source of Data for Policy. In: Macfarlane SB, AbouZahr C, editors. The Palgrave Handbook of Global Health Data Methods for Policy and Practice. London: Palgrave Macmillan UK (2019). p. 125–44. 10.1057/978-1-137-54984-6_7

[6] Setel P AbouZahr C Atuheire EB Bratschi M Cercone E Chinganya O Mortality Surveillance During the COVID-19 Pandemic. Bull World Health Organ (2020) 98(6):374. 10.2471/blt.20.263194 32514207 PMC7265935

[7] AbouZahr C . What Is the True Human Toll of COVID-19? For Better Answers to This Critical Question, Strengthen Civil Registration Systems (2020). Available from: https://www.vitalstrategies.org/what-is-the-true-human-toll-of-covid-19-for-better-answers-to-this-critical-question-strengthen-civil-registration-systems/ .

[8] EuroMOMO. European Mortality Monitoring Project. The European Monitoring of Excess Mortality for Public Health Action (EuroMOMO) Network (2020). Available from: https://www.euromomo.eu/ .

[9] UN Legal Identity Agenda. Impact of COVID19. Maintaining CRVS During the COVID-19 Pandemic (2020). Available from: https://unstats.un.org/legal-identity-agenda/COVID-19/ .

[10] Phillips DE AbouZahr C Lopez AD Mikkelsen L de Savigny D Lozano R Are Well Functioning Civil Registration and Vital Statistics Systems Associated with Better Health Outcomes? Lancet (2015) 386:1386–94. 10.1016/S0140-6736(15)60172-6 25971222

[11] Abouzahr C Azimi SY Bersales LGS Chandramouli C Hufana L Khan K Strengthening Civil Registration and Vital Statistics in the Asia-Pacific Region: Learning from Country Experiences. Asia Pacific Popul J (2012) 29(1):39–73. 10.18356/a906ccf5-en

[12] Higgins JP Green S . Cochrane Handbook for Systematic Reviews of Interventions. Chichester, England; Hoboken, NJ: Wiley-Blackwell (2011).

[13] Moher D Liberati A Tetzlaff J Altman DG . Preferred Reporting Items for Systematic Reviews and Meta-Analyses: the PRISMA Statement. Plos Med (2009) 6(7):e1000097. 10.1371/journal.pmed.1000097 19621072 PMC2707599

[14] Liberati A Altman DG Tetzlaff J Mulrow C Gøtzsche PC Ioannidis JPA The PRISMA Statement for Reporting Systematic Reviews and Meta-Analyses of Studies that Evaluate Health Care Interventions: Explanation and Elaboration. J Clin Epidemiol (2009) 62(10):e1–e34. 10.1016/j.jclinepi.2009.06.006 19631507

[15] Noyes J Booth A Moore G Flemming K Tunçalp Ö Shakibazadeh E . Synthesising Quantitative and Qualitative Evidence to Inform Guidelines on Complex Interventions: Clarifying the Purposes, Designs and Outlining Some Methods. BMJ Glob Health (2019) 4(Suppl. 1):e000893. 10.1136/bmjgh-2018-000893 PMC6350750 30775016

[16] Hong QN Pluye P Bujold M Wassef M . Convergent and Sequential Synthesis Designs: Implications for Conducting and Reporting Systematic Reviews of Qualitative and Quantitative Evidence. Syst Rev (2017) 6(1):61. 10.1186/s13643-017-0454-2 28335799 PMC5364694

[17] EPPI-Centre. Welcome to the EPPI-Reviewer 4 Gateway (2018). Available from: http://eppi.ioe.ac.uk/cms/Default.aspx?alias=eppi.ioe.ac.uk/cms/er4 .

[18] Hong Q Pluye P Fàbregues S Bartlett G Boardman F Cargo M Mixed Methods Appraisal Tool (MMAT), Version 2018. Canada: Canadian Intellectual Property Office (2018).

[19] Apland K Blitz B Hamylton C Lagaay M lakshman R Yarrow E . Birth Registration and Children's Rights: A Complex Story. Plan International (2014). Available from: https://plan-international.org/files/global/publications/campaigns/birth-registration-research-full-report.pdf .

[20] Fagernäs S . Protection Through Proof of Age. Birth Registration and Child Labor in Early 20th Century USA (2011).

[21] Fagernäs S . Papers, Please! The Effect of Birth Registration on Child Labor and Education in Early 20th Century USA. Explor Econ Hist (2014) 52:63–92. 10.1016/j.eeh.2013.09.002

[22] Ball J Butt L Beazley H . Birth Registration and Protection for Children of Transnational Labor Migrants in Indonesia. J Immigrant Refugee Stud (2017) 15:305–25. 10.1080/15562948.2017.1316533

[23] Seo BK . The Work of Inscription: Antenatal Care, Birth Documents, and Shan Migrant Women in Chiang Mai. Med Anthropol Q (2017) 31:481–98. 10.1111/maq.12342 27666134

[24] Chereni A . Underlying Dynamics of Child Birth Registration in Zimbabwe. Int J Child Rights (2016) 24:741–63. 10.1163/15718182-02404004

[25] Bowles J . Identifying the Rich: The Political Economy of Civil Registration in Tanzania (2018). Available from: http://barrett.dyson.cornell.edu/NEUDC/paper_269.pdf (Accessed October 15, 2019).

[26] Musarandega R . Integrated Human Rights and Poverty Eradication Strategy: The Case of Civil Registration Rights in Zimbabwe. Int Soc Sci J (2009) 60(197‐198):389–402. 10.1111/j.1468-2451.2010.01728.x 20726138

[27] Kusumaningrum S Bennouna C Siagian C Agastya NLPM . Back to What Counts: Birth and Death in Indonesia. Jakarta, Indonesia: The Center on Child Protection Universitas Indonesia (PUSKAPA) in Collaboration with the Ministry of National Development Planning (BAPPENAS) and Kolaborasi Masyarakat dan Pelayanan untuk Kesejahteraan-KOMPAK (2016).

[28] Corbacho A Brito S Osorio Rivas R . Birth Registration and the Impact on Educational Attainment. IDB Working Paper Series (2012). Contract No: IDB-WP-345.

[29] Jeong J Bhatia A Fink G . Associations Between Birth Registration and Early Child Growth and Development: Evidence from 31 Low- and Middle-Income Countries. BMC Public Health (2018) 18:673. 10.1186/s12889-018-5598-z 29848302 PMC5977554

[30] Comandini O Cabras S Marini E . Birth Registration and Child Undernutrition in Sub-Saharan Africa. Public Health Nutr (2016) 19(10):1757–67. 10.1017/s136898001500333x 26669828 PMC10271021

[31] Brito S Corbacho A Osorio R . Does Birth Under-Registration Reduce Childhood Immunization? Evidence from the Dominican Republic. Health Econ Rev (2017) 7:14. 10.1186/s13561-017-0149-3 28337738 PMC5364131

[32] Goldsen J Bryan AEB Kim H-J Muraco A Jen S Fredriksen-Goldsen KI . Who Says I Do: The Changing Context of Marriage and Health and Quality of Life for LGBT Older Adults. Geront (2017) 57:S50–S62. 10.1093/geront/gnw174 PMC5241756 28087795

[33] Raifman J Moscoe E Austin SB McConnell M . Difference-in-Differences Analysis of the Association Between State Same-Sex Marriage Policies and Adolescent Suicide Attempts. JAMA Pediatr (2017) 171(4):350–6. 10.1001/jamapediatrics.2016.4529 28241285 PMC5848493

[34] Riggle EDB Wickham RE Rostosky SS Rothblum ED Balsam KF . Impact of Civil Marriage Recognition for Long-Term Same-Sex Couples. Sex Res Soc Pol (2017) 14:223–32. 10.1007/s13178-016-0243-z

[35] Maduekwe NI Banjo OO Sangodapo MO . Data for the Sustainable Development Goals: Metrics for Evaluating Civil Registration and Vital Statistics Systems Data Relevance and Production Capacity, Illustrations with Nigeria. Soc Indic Res (2017) 140:101–24. 10.1007/s11205-017-1760-8

[36] Cobos Muñoz D de Savigny D Sorchik R Bo KS Hart J Kwa V Better Data for Better Outcomes: the Importance of Process Mapping and Management in CRVS Systems. BMC Med (2020) 18(1):67. 10.1186/s12916-020-01522-z 32146901 PMC7061473

[37] Shattuck L . The Shattuck Report (1850). Available from: https://biotech.law.lsu.edu/cphl/history/books/sr (Accessed December 15, 2020).

[38] Hawke A . Birth Registration: Right from the Start. Florence, Italy: Innocenti Digest (2002).

[39] Szreter S . The Right of Registration: Development, Identity Registration, and Social Security-A Historical Perspective. World Develop (2007) 35(1):67–86. 10.1016/j.worlddev.2006.09.004

[40] Mathers CD Fat DM Inoue M Rao C Lopez AD . Counting the Dead and what They Died from: An Assessment of the Global Status of Cause of Death Data. Bull World Health Organ (2005) 83:171–7. 15798840 PMC2624200

[41] Ruzicka L Kane P Wunsch G . Differential Mortality: Methodological Issues and Biosocial Factors. Oxford University Press (1995). Available from: https://econpapers.repec.org/bookchap/oxpobooks/9780198288824.htm .

[42] AbouZahr C Cleland J Coullare F Macfarlane SB Notzon FC Setel P The Way Forward. Lancet (2007) 370(9601):1791–9. 10.1016/s0140-6736(07)61310-5 18029003

[43] Fagernäs S Odame J . Birth Registration and Access to Health Care: An Assessment of Ghana's Campaign Success. Bull World Health Organ (2013) 91(6):459–64. 10.2471/blt.12.111351 24052683 PMC3777139

[44] Corrêa G Verstraete P Soundardjee R Shankar M Paterson C Hampton L Immunization Programmes and Notifications of Vital Events. Bull World Health Organ (2019) 97(4):306–8. 10.2471/blt.18.210807 30940988 PMC6438247

[45] Driscoll J . Seeing Like A Subaltern: State-Building, Census Taking, & the Political Economy of Population Counts. Social Science Research Network (2016). 10.2139/SSRN.2825378

[46] Mills S Amponsah D . Economic Analysis of Producing Vital Statistics Using Civil Registration Data in Lao People's Democratic Republic. J Health Popul Nutr (2019) 38(Suppl. 1):20. 10.1186/s41043-019-0184-2 31627762 PMC6800530

[47] Nichols EK Ragunanthan NW Ragunanthan B Gebrehiwet H Kamara K . A Systematic Review of Vital Events Tracking by Community Health Agents. Glob Health Action (2019) 12(1):1597452. 10.1080/16549716.2019.1597452 31179875 PMC6566585

[48] Lu T-H Lee M-C Chou M-C . Accuracy of Cause-Of-Death Coding in Taiwan: Types of Miscoding and Effects on Mortality Statistics. Int J Epidemiol (2000) 29(2):336–43. 10.1093/ije/29.2.336 10817134

[49] World Health Organization. Improving Mortality Statistics Through Civil Registration and Vital Statistics Systems: Strategies for Country and Partner Support - Outcome of a Technical Meeting. Geneva, Switzerland: World Health Organization (2014).

[50] Sumner C Kusumaningrum S . Indonesia’s Missing Millions: Erasing Discrimination in Birth Certification in Indonesia (CGD Policy Paper 064) (2015). Washington, DC: Center for Global Development.

[51] Ordóñez Bustamante D Bracamonte Bardález P . El registro de nacimientos: Consecuencias en relación al acceso a derechos y servicios sociales y a la implementación de programas de reducción de pobreza en 6 países de Latinoamérica (2006). Available from: https://publications.iadb.org/handle/11319/6309 (Accessed January 14, 2020).

[52] Carter Center. Voter Identification Requirements and Public International Law: An Examination of Africa and Latin America. Research study. Atlanta: The Carter Center (2013).

[53] UNICEF. The "Rights" Start to Life: A Statistical Analysis of Birth Registration. Unicef (2005). New York.

[54] Dahan M Hanmer L . The Identification for Development (ID4D) Agenda: Its Potential for Empowering Women and Girls (2015). The World Bank. Available from: https://openknowledge.worldbank.org/handle/10986/22795 (Accessed January 10, 2020).

[55] Plan. Birth Registration in Emergencies: A Review of Best Practices in Humanitarian Action (2014). Report No: ISBN: 978-1-906273-63-7.

[56] Castan M Gerber P Gargett A . Indigenous Australians' Access to Birth Registration Systems: A Breach of International Human Rights Law? Aust J Hum Rights (2011) 17:55–89. 10.1080/1323238x.2011.11910895

[57] Calabro A . Registering the Births of Indigenous Australians: Has New South Wales Got it Right? Univ New South Wales L J (2013) 36:809–38. 10.3316/informit.736228515903213

[58] Gupta A . Hazme Visible Indigenous Children's Rights in Chiapas. DePaul J Soc Justice (2012) 5:379–91.

[59] Sanders C Burnett K . A Case Study in Personal Identification and Social Determinants of Health: Unregistered Births Among Indigenous People in Northern Ontario. Int J Environ Res Public Health (2019) 16, 567. 10.3390/ijerph16040567 PMC6406902 30781459

[60] Norwegian Refugee Council. Update on Marriage Registration for Refugees from Syria. Understanding the Procedures and Identifying the Challenges Faced by Refugees when Registering Marriages in Lebanon (2016). Available from: https://www.nrc.no/globalassets/pdf/reports/update-onmarriage-registration-for-refugees-from-syria.pdf (Accessed July 15, 2020).

[61] UN. Report of the Executive Committee of the Programme of the United Nations High Commissioner for Refugees on the Fifty-Fourth Session (2013). (29 September-3 October 2003).

[62] Cobos Muñoz D Abouzahr C de Savigny D . The ‘Ten CRVS Milestones' Framework for Understanding Civil Registration and Vital Statistics Systems. BMJ Glob Health (2018) 3(2):e000673. 10.1136/bmjgh-2017-000673 PMC5873547 29607102

[63] Niamba L . Civil Registration and Vital Statistics (CRVS) Systems in the Face of the COVID-19 Pandemic: A Literature Review. CRVS Working Paper Series, 3 (2021).

[64] AbouZahr C Bratschi MW Cercone E Mangharam A Savigny Dd. Dincu I The COVID-19 Pandemic: Effects on Civil Registration of Births and Deaths and on Availability and Utility of Vital Events Data. Am J Public Health (2021) 111(6):1123–31. 10.2105/ajph.2021.306203 33856881 PMC8101592

[65] SDD. Impact of the Covid-19 Pandemic on Operations of National Civil Registration and Vital Statistics (CRVS) Systems: 2020. Noumea, New Caledonia: Pacific Community (2020). p. 30.

[66] Mills S Lee JK Rassekh BM . Benefits of Linking Civil Registration and Vital Statistics with Identity Management Systems for Measuring and Achieving Sustainable Development Goal 3 Indicators. J Health Popul Nutr (2019) 38(1):18. 10.1186/s41043-019-0178-0 31627734 PMC6800484

[67] Fu H Schweinfest S . COVID-19 Widens Gulf of Global Data Inequality, While National Statistical Offices Step Up to Meet New Data Demands. Data Blog (2020). [Internet].

