# Supplementary material for: Addressing the Evidence Gap in the Economic and Social Benefits of Civil Registration and Vital Statistics Systems: A Systematic Review
Source: Public Health Rev. 2022 Jul 8;43:1604560. doi: 10.3389/phrs.2022.1604560 (PMC9330020; doi:10.3389/phrs.2022.1604560)
Supplement: Supplementary file 1 [file DataSheet3.docx]

Supplementary Fila S3. Systematic review search strategy. Addressing the evidence gap in the economic and social benefits of Civil Registration and Vital Statistics Systems: A Systematic Review, 2021. (Systematic review, Asia, America, Africa and Europe, 1910–2019).

Addressing the Evidence Gap in the economic and social benefits of Civil Registration and Vital Statistics Systems:

A Systematic Review

# Search Strategy

## Search methods for identification of studies:

An extensive and comprehensive search of literature will be conducted. The appropriate research strategy will be developed by an independent information specialist, in consultations with the review team. The strategy with the selected terms, will be developed and tested prior to the final search. The terms and search filters will comprise the title, abstract, and keywords, in combination with the subject headings of databases (e.g. MeSH).

We will include studies published until the search date from the following sources:

#### Electronic databases:

- - Cochrane Central Register of Controlled Trials (CENTRAL)
  - MEDLINE (Ovid)
  - Excerpta Medical Database (EMBASE) (Ovid)
  - PubMed
  - Econlit
  - Scopus
  - ELDIS
  - Global health library (WHO)
  - WHO IMSEAR (WHO Index Medicus South East Asian Region) and IndMED
  - Electronic databases for grey literature
  - Ebsco Discovery - Econlit, Repec, World Bank e-library, Academic Search Complete
  - Web of Science
  - CAB Global Health
  - Epistemonikos

#### Subject-specific databases and National, regional - literatures not indexed in major medical and health databases

- - Relevant web-based databases and repositories of relevant institutions and organizations (e.g. The World Bank Group, ID4D, World Health Organization (WHO), UNECA or ESCAP, IDRC or Bloomberg D4H resource gateway)

#### Other resources

- - Conference abstract archives from systematic review and library methods conferences: Canadian Health Libraries Association (Canada), Cochrane Colloquium, etc.
  - In addition to searching electronic databases, and in order to identify further appropriate publications, we will contact individual researchers, experts working in the field and authors who mainly work on CRVS field, to address whether any relevant manuscripts are in preparation or in press.
  - General search engines (google and google scholar)

#### Citation indexes

- - The search strategy will be iterative, in that references of included studies will be searched for additional references.

# Ovid MEDLINE(R) and Epub Ahead of Print, In-Process & Other Non-Indexed Citations, Daily and Versions(R) <1946 to June 27, 2019> Searched 30th June 2019

1 *records/ or *birth certificates/ or *death certificates/ or *cause of death/ or *registries/ or *vital statistics/ or *population surveillance/ (62454)

2 (((civil* or citizen* or population or national or identity or identities) adj2 (registr* or register* or record* or document* or enrol* or status)) or crvs or (vital adj (statistics or event*))).ti,ab,kw. (33928)

3 (((national or population or civil or citizen* or legal) adj3 ((identit* or identif* or authenticat*) adj2 (system or systems))) or "health metrics network").ti,ab,kw. (137)

4 ((birth* or natality or death* or mortality or marriage* or divorce* or adoption*) adj3 (registr* or notif* or report* or certif* or survey* or surveillance)).ti,ab,kw. (41830)

5 Marital Status/sn or Divorce/sn or Marriage/sn or Adoption/sn (2901)

6 or/1-5 (129662)

7 *"costs and cost analysis"/ or *"cost allocation"/ or *cost-benefit analysis/ or *"cost control"/ or *"cost savings"/ or *direct service costs/ or *health expenditures/ (29444)

8 (cost* or economic* or budget* or financ* or expense* or expenditure* or saving*).ti,ab,kw. (922786)

9 *policy/ or *public policy/ or *health policy/ or *social control, formal/ or *government regulation/ or *civil rights/ or *social justice/ or *human rights/ or *patient rights/ or *women's rights/ or *patient advocacy/ or *policy making/ or *health services accessibility/ or *healthcare disparities/ or *global health/ or *"Outcome Assessment (Health Care)"/ or *decision making/ (221426)

10 (policy or policies or policy-making or ((civil or patient* or women* or human) adj right*) or "social justice" or "global health" or ((access* or disparit* or inequalit*) adj2 (health or healthcare or service* or welfare or opportunit*))).ti,ab,kw. (294809)

11 or/7-10 (1296605)

12 6 and 11 (16373)

13 (news or comment or editorial).pt. or comment on.cm. (1308672)

14 12 not 13 (16047)

# Embase Classic+Embase <1947 to 2019 June 28> Searched 1^st^ July 2019

1 vital statistics/ or birth certificate/ or death certificate/ or cause of death/ or registration/ or register/ or civil registration/ or disease registry/ or "civil registration and vital statistics system"/ or medical information system/ or certification/ (323594)

2 (((civil* or citizen* or population or national or identity or identities) adj2 (registr* or register* or record* or document* or enrol* or status)) or crvs or (vital adj (statistics or event*))).ti,ab,kw. (48750)

3 (((national or population or civil or citizen* or legal) adj3 ((identit* or identif* or authenticat*) adj2 (system or systems))) or "health metrics network").ti,ab,kw. (174)

4 ((birth* or natality or death* or mortality or marriage* or divorce* or adoption*) adj3 (registr* or notif* or report* or certif* or survey* or surveillance)).ti,ab,kw. (59682)

5 or/1-4 (392416)

6 (cost* or economic* or budget* or financ* or expense* or expenditure* or saving*).ti,ab,kw. (1226470)

7 economic evaluation/ or "cost benefit analysis"/ or "cost control"/ or "cost effectiveness analysis"/ or "cost minimization analysis"/ or "cost utility analysis"/ (278002)

8 "health care cost"/ or health care financing/ or resource allocation/ (207278)

9 (policy or policies or policy-making or ((civil or patient* or women* or human) adj right*) or "social justice" or "global health" or ((access* or disparit* or inequalit*) adj2 (health or healthcare or service* or welfare or opportunit*))).ti,ab,kw. (375999)

10 policy/ or health care policy/ or community assessment/ or civil rights/ or human rights/ or patient right/ or reproductive rights/ or social justice/ or women's rights/ or health care access/ or health disparity/ (385040)

11 or/6-10 (1858758)

12 5 and 11 (47548)

13 (editorial or note).pt. (1383080)

14 12 not 13 (44387)

15 limit 14 to exclude medline journals (3773)
