# Supplementary material for: Addressing the Evidence Gap in the Economic and Social Benefits of Civil Registration and Vital Statistics Systems: A Systematic Review
Source: Public Health Rev. 2022 Jul 8;43:1604560. doi: 10.3389/phrs.2022.1604560 (PMC9330020; doi:10.3389/phrs.2022.1604560)
Supplement: Supplementary file 3 [file DataSheet1.docx]

Supplementary File S1 Quality Appraisal

Listing the quality appraisal ratings of each study using Mixed Methods Appraisal Tool (18). Addressing the evidence gap in the economic and social benefits of Civil Registration and Vital Statistics Systems: A Systematic Review, 2021. (Systematic review, Asia, America, Africa and Europe, 1910–2019).

|  | | Criteria from the Mixed Methods Appraisal Tool | | | | | | | | | | | | | | | | | | | | |  |
| --- | --- | --- | --- | --- | --- | --- | --- | --- | --- | --- | --- | --- | --- | --- | --- | --- | --- | --- | --- | --- | --- | --- | --- |
| First Author, Year | **Study Type** | **1. Qualitative** | | | | | | **3. Quantitative nonrandomized** | | | | | **4. Quantitative descriptive** | | | | | **5. Mixed methods: Methodological quality criteria** | | | | | **QA score** |
|  |  | 1.1 | 1.2 | 1.3 | 1.4 | 1.5 | 3.1 | | 3.2 | 3.3 | 3.4 | 3.5 | 4.1 | 4.2 | 4.3 | 4.4 | 4.5 | 5.1 | 5.2 | 5.3 | 5.4 | 5.5 |  |
| Apland 2014 | MM | 1 | 1 | 1 | 1 | 1 |  | |  |  |  |  | 0 | 1 | 1 | 0 | 1 | 1 | 1 | 1 | 0 | 1 | 5,3,4 |
| Ball 2017 | QL | 1 | 1 | 1 | 0 | 1 |  | |  |  |  |  |  |  |  |  |  |  |  |  |  |  | 4 |
| Bowles 2018 | QT |  |  |  |  |  | 1 | | 1 | 0 | 1 | 1 |  |  |  |  |  |  |  |  |  |  | 4 |
| Brito 2017 | QT |  |  |  |  |  | 1 | | 1 | 1 | 1 | 1 |  |  |  |  |  |  |  |  |  |  | 5 |
| Chereni 2016 | MM | 1 | 1 | 1 | 1 | 1 |  | |  |  |  |  | 1 | 1 | 1 | 0 | 1 | 1 | 1 | 1 | 1 | 1 | 5,4,4 |
| Comandini 2015 | QT |  |  |  |  |  | 1 | | 1 | 1 | 1 | 1 |  |  |  |  |  |  |  |  |  |  | 5 |
| Corbacho 2012 | QT |  |  |  |  |  | 1 | | 1 | 1 | 1 | 1 |  |  |  |  |  |  |  |  |  |  | 5 |
| Fagernäs 2014 | QT |  |  |  |  |  | 1 | | 0 | 1 | 1 | 1 |  |  |  |  |  |  |  |  |  |  | 4 |
| Fagernäs 2011 | QT |  |  |  |  |  | 1 | | 0 | 1 | 1 | 1 |  |  |  |  |  |  |  |  |  |  | 4 |
| Goldsen 2017 | QT |  |  |  |  |  | 1 | | 1 | 1 | 1 | 1 |  |  |  |  |  |  |  |  |  |  | 5 |
| Jeong 2018 | QT |  |  |  |  |  | 1 | | 1 | 1 | 1 | 1 |  |  |  |  |  |  |  |  |  |  | 5 |
| Kusumaningrum 2016 | MM | 1 | 1 | 0 | 0 | 1 |  | |  |  |  |  | 1 | 1 | 1 | 0 | 0 | 1 | 1 | 1 | 0 | 0 | 3,3,4 |
| Maduekwe 2018 | MM | 1 | 1 | 1 | 1 | 1 |  | |  |  |  |  | 1 | 1 | 1 | 0 | 0 | 1 | 1 | 1 | 0 | 0 | 5,3,3 |
| Musarandega 2009 | QL | 1 | 1 | 1 | 0 | 1 |  | |  |  |  |  |  |  |  |  |  |  |  |  |  |  | 4 |
| Phillips 2015 | QT |  |  |  |  |  | 0 | | 1 | 1 | 0 | 1 |  |  |  |  |  |  |  |  |  |  | 3 |
| Raifman 2019 | QT |  |  |  |  |  | 1 | | 1 | 1 | 1 | 1 |  |  |  |  |  |  |  |  |  |  | 5 |
| Riggle 2017 | QT |  |  |  |  |  | 1 | | 1 | 1 | 1 | 1 |  |  |  |  |  |  |  |  |  |  | 5 |
| Seo 2017 | MM | 1 | 1 | 1 | 1 | 0 |  | |  |  |  |  | 0 | 0 | 0 | 1 | 0 | 1 | 1 | 1 | 0 | 0 | 4,1,3 |

1 = a score of ‘yes’; 0 = a score of ‘no’ or ‘can’t tell’

MM= Mixed Methods, QL = Qualitative, QT= Quantitative, QA = Quality Appraisal

QA score 5= strong, QA score 4 and 3= moderate, QA score 2 and 1= weak

| **Criteria within the Mixed Methods Appraisal Tool** | |
| --- | --- |
| **Category of study designs** | **Methodological quality criteria** |
| 1. Qualitative research approach | 1.1. Is the qualitative approach appropriate to answer the research question? |
|  | 1.2. Are the qualitative data collection methods adequate to address the research question? |
|  | 1.3. Are the findings adequately derived from the data? |
|  | 1.4. Is the interpretation of results sufficiently substantiated by data? |
|  | 1.5. Is there coherence between qualitative data sources, collection, analysis and interpretation? |
| 2. Quantitative randomized controlled trials | 2.1. Is randomization appropriately performed? |
|  | 2.2. Are the groups comparable at baseline? |
|  | 2.3. Are there complete outcome data? |
|  | 2.4. Are outcome assessors blinded to the intervention provided? |
|  | 2.5 Did the participants adhere to the assigned intervention? |
| 3. Quantitative nonrandomized | 3.1. Are the participants representative of the target population? |
|  | 3.2. Are measurements appropriate regarding both the outcome and intervention (or exposure)? |
|  | 3.3. Are there complete outcome data? |
|  | 3.4. Are the confounders accounted for in the design and analysis? |
|  | 3.5. During the study period, is the intervention administered (or exposure occurred) as intended? |
| 4. Quantitative descriptive | 4.1. Is the sampling strategy relevant to address the research question? |
|  | 4.2. Is the sample representative of the target population? |
|  | 4.3. Are the measurements appropriate? |
|  | 4.4. Is the risk of nonresponse bias low? |
|  | 4.5. Is the statistical analysis appropriate to answer the research question? |
| 5. Mixed methods | 5.1. Is there an adequate rationale for using a mixed methods design to address the research question? |
|  | 5.2. Are the different components of the study effectively integrated to answer the research question? |
|  | 5.3. Are the outputs of the integration of qualitative and quantitative components adequately interpreted? |
|  | 5.4. Are divergences and inconsistencies between quantitative and qualitative results adequately addressed? |
|  | 5.5. Do the different components of the study adhere to the quality criteria of each tradition of the methods involved? |
